# High antinuclear antibody titer is associated with increased mortality risk in patients with idiopathic pulmonary fibrosis

**DOI:** 10.1007/s10238-024-01447-4

**Published:** 2024-07-28

**Authors:** Shimon Izhakian, May Igawa, Liora Chen Zion, Ori Mekiten, Lev Freidkin, Dror Rosengarten, Moshe Heching, Mordechai Reuven Kramer

**Affiliations:** 1 Pulmonary Institute, Rabin Medical Center–Beilinson Hospital, 39 Jabotinski St., 4941492 Petach Tikva, Israel; 2 Faculty of Medicine, Tel Aviv University, 6997801 Tel Aviv, Israel; 3 The Adelson School of Medicine, Ariel University, 4076414 Ariel, Israel

**Keywords:** Idiopathic pulmonary fibrosis, Antinuclear antibody, Mortality, Survival, Lung fibrosis

## Abstract

Idiopathic pulmonary fibrosis (IPF) is a diagnosis of exclusion, requiring that potential etiologies of interstitial lung disease be ruled out. Antinuclear antibody (ANA) testing is commonly performed in individuals with IPF, but the clinical significance of ANA positivity remains uncertain. A retrospective search identified 161 patients diagnosed with IPF between May 2010 and January 2021. Data on ANA titers at the time of diagnosis were available in all cases. Mean age of the patients was 66.4 ± 9.6 years; 70.8% were male. ANA titers were high (≥ 1:160) in 25.4% of the cohort. Baseline characteristics were comparable between those with high and low ANA titers. During follow-up (median 28 months), 93 patients (57%) died. On Cox proportional-hazards analysis with lung transplantation entered as a competing risk and adjusting for potential confounders (age, sex, and baseline forced vital capacity and diffusing lung capacity for carbon monoxide), ANA ≥ 1:160, as a dichotomized variable, was significantly associated with case-specific mortality (HR 2.25, 95% CI 1.14−4.42, *P* = 0.02) and older age (for each 10-year increment, HR 1.55, 95% CI 1.07−2.25, *P* = 0.02). High ANA titers appear to be associated with increased mortality in IPF. This finding emphasizes the potential prognostic value of ANA testing. Further studies are needed to validate these findings and explore their implications for patient management.

## Introduction

Idiopathic pulmonary fibrosis (IPF) is a progressive fibrosing interstitial lung disease (ILD) of unknown origin, primarily affecting older adults and confined to the lungs [1, 2]. It is characterized by a specific radiological/histopathological pattern of usual interstitial pneumonia. Given that connective tissue disease (CTD) and many other fibrotic ILDs of various etiologies exhibit similar radiologic and histopathologic findings to IPF, establishing the diagnosis of IPF depends on their exclusion. Consequently, in patients suspected of having IPF, consensus guidelines recommend screening for a panel of autoantibodies, including antinuclear antibodies (ANA) [1, 2]. Although high ANA titers (≥ 1:160) have been observed in patients with CTD and other fibrotic ILDs, their presence alone, without clinical features of CTD and other autoantibodies, is insufficient to establish a diagnosis of CTD [3—7]. Therefore, IPF can be diagnosed in cases of a fibrotic ILD with a usual interstitial pneumonia pattern, even in the presence of a high ANA titer. However, the clinical significance of ANA positivity remains uncertain.

Published data on the association between ANA titer and IPF prognosis are limited [4]. However, based on our clinical experience, we have observed that IPF patients with high ANA titers often exhibit a more aggressive disease phenotype. Therefore, we hypothesized that clinical features and prognosis may vary among IPF patients based on their ANA titers.

The aim of this study was to compare demographics, comorbidities, pulmonary function, and mortality between individuals with IPF with high (≥ 1:160) or low (< 1:160) ANA titers, in order to further investigate the potential impact of ANA positivity on disease outcomes.

## Materials and methods

### Study population and design

The cohort consisted of patients diagnosed with IPF at the Pulmonary Institute of a tertiary university medical center in Israel between May 2010 and January 2021. Patients were identified by retrospective search of the electronic database. The diagnosis of IPF was established based on the decision of a multidisciplinary discussion (MDD) involving pulmonologists, radiologists, and pathologists to ensure a comprehensive evaluation and accurate diagnosis. The MDD considered clinical symptoms, an autoimmune antibody panel, and high-resolution computed tomography findings consistent with IPF and lung biopsy if needed. When imaging results were inconclusive, lung biopsies were performed. Patients with alternative etiologies of fibrotic ILD, such as CTD, occupational exposure, hypersensitivity pneumonitis, and drug exposure, were excluded from the analysis [1, 2]. Patients with missing data on ANA titers at the time of diagnosis were excluded as well.

Data were collected for all participants on age, sex, comorbidities, pulmonary function test parameters, ANA titers, and survival status at the end of follow-up. Patients were followed until death, lung transplantation, or completion of the study in March 2022.

### ANA evaluation

ANA evaluation was performed using immunofluorescence on either the HELIOS (ASKU Diagnostics GMBH, Germany) or PhD (BIORAD, San Francisco, CA, USA) automated system. Slides were sourced from the Hep-2000 cell line (Immuno Concepts, Sacramento, CA USA or AESKU Diagnostics). Samples were tested at dilutions of 1:40, 1:80, and 1:160. A positive result was defined as the observation of fluorescence intensity at a dilution of 1:160. Fluorescence intensity higher than 1:160 was considered > 160.

### Statistical analysis

Descriptive data were summarized as mean and standard deviation or number and percentage. Data were analyzed for the entire cohort and compared between two subgroups, stratified according to high (≥ 1:160) ANA titers and low (< 1:160) or negative ANA titers. Chi-square test was used to assess categorical variables, and Student’s t-test, for continuous variables. *P* values < 0.05 were considered statistically significant.

Risk factors for death or lung transplantation in the entire cohort were assessed using competing risk regression analysis with the Fine and Gray model. A Cox proportional-hazards model with competing risk regression was applied to examine the effects of covariates such as age, gender, forced vital capacity (FVC), and diffusing lung capacity for carbon monoxide (DLCO) on the association of high ANA titer (≥ 1:160) with case-specific mortality. In this model, lung transplantation was considered a competing risk for mortality.

Statistical analyses were conducted using SAS software, version 9.2 (SAS Institute Inc.).

## Results

### Patient characteristics

**Fig. 1:**
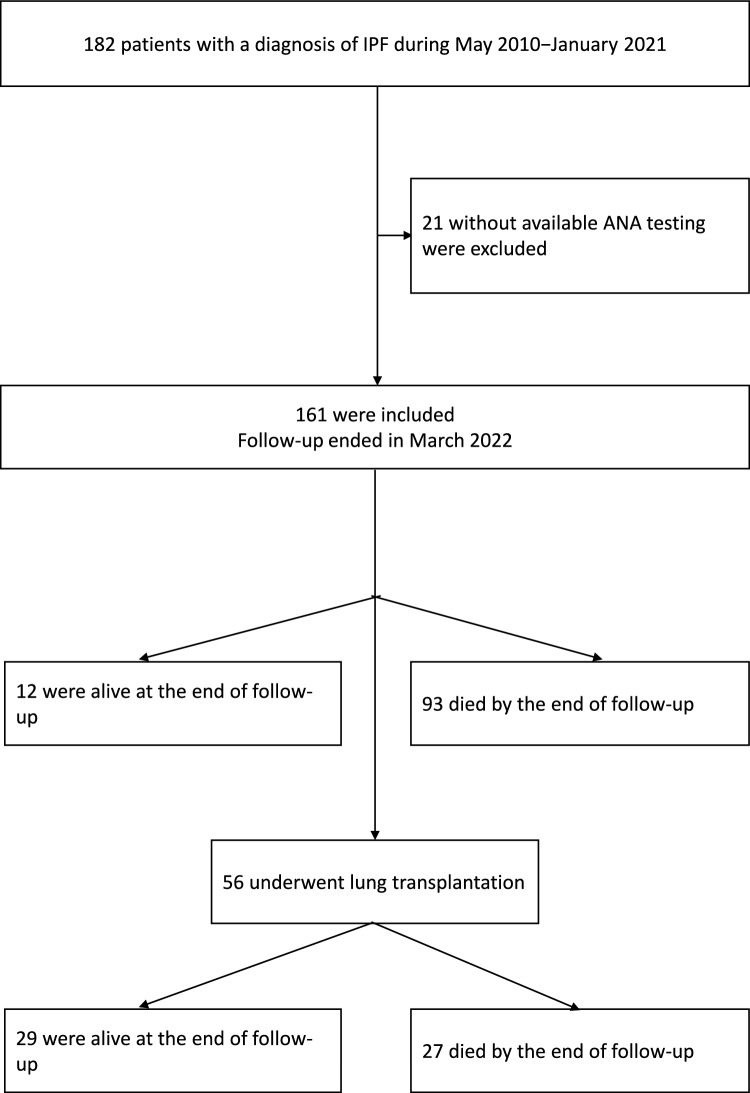
Flowchart of patient selection and survival. Abbreviations: IPF, idiopathic pulmonary fibrosis; ANA, antinuclear antibody

During the study period, 182 patients were diagnosed with IPF of whom 161 completed ANA testing and were included in the study (Fig. 1). Mean age of the cohort was 66.4 ± 9.6 years; 70.8% were male. High titers were found in 41 patients (25.5%) and low titers in 120 (74.5%). In the low-titer group, 69 patients (57.5%) had a positive ANA test, and 51 had a negative ANA test. Table 1 presents the baseline characteristics of the entire sample and stratified by high/low ANA titer. There were no significant between-group differences in demographic characteristics, prevalence of comorbidities, and pulmonary function test parameters.

**Table 1:** Baseline characteristics of 161 patients with IPF, whole cohort and by ANA titer

Characteristic	Entire sample	ANA titer ≥ 160 (n = 41)	ANA titer < 160 (n = 120)	*P* value*
Age (yrs)	66.4 ± 9.6	66.1 ± 9.9	67.0 ± 8.6	0.80
Male sex	114 (70.8%)	28 (68.3%)	86 (71.1%)	0.84
Patients on antifibrotic therapy	1223 (76.4%)	33 (80.4%)	90 (75.0%)	0.37
*Comorbidities*				
Ischemic heart disease	52 (32.3%)	15 (36.6%)	37 (308%)	0.56
Hypertension	72 (44.7%)	16 (39.0%)	56 (46.7%)	0.46
Cerebrovascular disease	6 (3.7%)	2 (4.9%)	4 (3.3%)	0.64
Hyperlipidemia	98 (60.9%)	25 (60.9%)	73 (60.8%)	1.0
Diabetes mellitus	60 (37.3%)	14 (34.1%)	46 (38.3%)	0.71
GERD	47 (29.2%)	11 (26.8%)	36 (30.0%)	0.84
*Lung function test results at IPF diagnosis*				
FVC (% of predicted value)	63.4 ± 18.2	59.6 ± 18.1	64.8 ± 18.1	0.13
DLCO (% of predicted value)	45.0 ± 15.1	43.6 ± 13.6	45.5 ± 15.8	0.87
*At end of follow-up*				
FVC (% of predicted value)	54.5 ± 20.8	46.3 ± 20.9	57.1 ± 20.1	0.005
DLCO (% of predicted value)	32.9 ± 15.2	30.0 ± 15.4	33.8 ± 15.1	0.20
Delta FVC	10.0 ± 16.7	11.8 ± 12.6	9.4 ± 17.9	0.41
Delta DLCO	13.2 ± 14.5	15.9 ± 15.7	12.3 ± 14.1	0.36

The data are presented as mean ± standard deviation or number (percentage) of presented cases

^*^Statistical comparison between groups with ANA titers ≥ 160 and < 160

Abbreviations: IPF, idiopathic pulmonary fibrosis; ANA, antinuclear antibody; GERD, gastroesophageal reflux disease; FVC, forced vital capacity; DLCO, diffusing lung capacity for carbon monoxide

During the follow-up period (median 28 months), anti-fibrotic treatment was administered to 123 patients (87.0%). (Anti-fibrotic agents became available for use in Israel in 2015.) The use of anti-fibrotic agents did not differ significantly between patients with high and low ANA titers (*P* = 0.37). Lung transplantation was performed during follow-up in 56 patients (30.4%), 7 with high ANA titers and 49 with low ANA titers, accounting for 17.1% and 40.8% of the respective groups.

### Survival (Fig. 2)

**Fig. 2:**
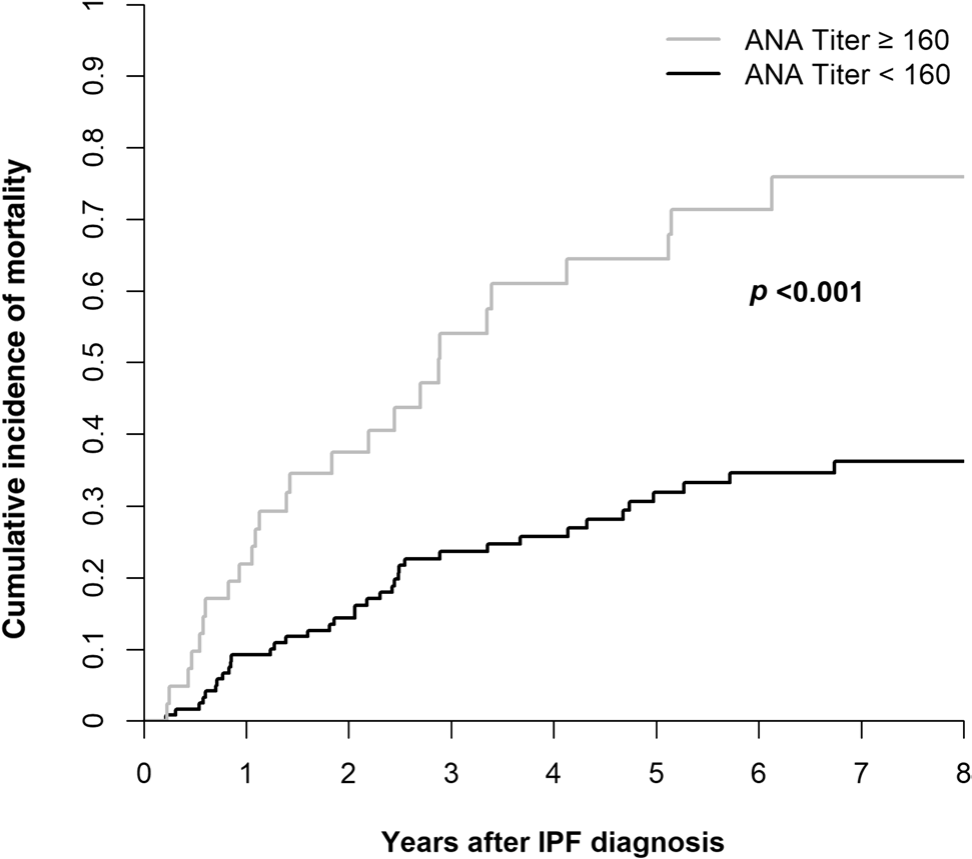
Cumulative incidence of survival in patients with IPF, stratified by high (≥ 160) and low (< 160) ANA titers at diagnosis

During the follow-up period, 93 (57.8%) patients died, including 66 (41.0% of the total) who had not received a transplant and 27 (16.7% of the total) who had undergone lung transplantation. Of the 68 patients (42.2%) who were alive until the end of follow-up, 29 had received a lung transplant and 39 were either awaiting a lung transplant or were not candidates for transplantation. The corresponding mortality rates for patients with high and low ANA titers were 68.3% and 54.1% (*P* < 0.001).

In the Cox proportional-hazards model described above, high ANA titers, when treated as a dichotomized variable, remained strongly associated with case-specific mortality [hazard ratio (HR) 2.25, 95% confidence interval (CI) 1.14−4.20, *P* = 0.02], along with older age (for each 10-year increment, HR 1.55, 95% CI 1.07−1.55, *P* = 0.02).

### Lung function tests

While no statistically significant differences were observed between the high and low ANA titer groups in baseline FVC (*P* = 0.13) and DLCO (*P* = 0.87), there is a trend towards a higher FVC in the ANA titer < 160 group. Additionally, no significant differences were found in changes in these parameters from baseline to the end of follow-up (FVC, *P* = 0.41, and DLCO, *P* = 0.36).

## Discussion

The present study of 161 patients with IPF showed that 25.5% had a high ANA titer (≥ 1:160). In other studies of patients with IPF, the proportion of positive ANA testing ranged from 26.3% to 41.4% [3—7]. The variability in positive ANA serologies among the studies may be explained by the different cut-off titers used to define a positive ANA test, from as low as 1:40 [3, 5] and 1:80 [6] up to 1:160 [4] and 1:320 [7].

Positive ANA tests have been reported in healthy individuals [8, 9]; about 5% are found to have a titer of 1:160 or more [8]. The known increase in the prevalence of positive ANA serology with age in the general population [8, 9] may contribute to the higher proportion of ANA positivity in patients with IPF, a disease generally diagnosed in older age [10]. It is noteworthy that in the general population, healthy females are more likely to have positive ANA serology than males [11], whereas we observed a high prevalence of positive ANA in our predominantly male IPF cohort.

Mortality in the present study was higher in patients with IPF who had a high (≥ 1:160) ANA titer than in those with a low ANA titer. This finding contrasts with an earlier study of 58 patients with IPF in which no association was noted between a high ANA titer (≥ 160) and survival [4]. However, our study included a larger sample and accounted for potential confounders, probably yielding more robust results due to the greater statistical power.

An association between a high ANA titer and poor IPF prognosis is in line with the proposed role of immune cells in the pathogenesis of IPF. Pulmonary fibrosis is hypothesized to result from repeated epithelial lung injury, leading, in susceptible individuals, to aberrant repair and the formation of fibrotic tissue [12, 13]. Immune cells are thought to migrate to the site of epithelial injury where they facilitate repair. The presence of serum ANA autoantibodies in high titers may serve as an indicator of immune system dysfunction, suggesting that pathological fibrotic repair is catalyzed through an autoantibody-mediated process, ultimately accelerating the disease pathogenesis.

Identifying measurable serum biomarkers, such as ANA, that predict IPF pathophysiology may help guide treatment approaches. Prospective studies are needed to thoroughly investigate the relationship of ANA titers with outcomes in IPF, as well as their association with treatment response. The ease and speed with which biomarkers can be measured will determine their applicability within a clinical setting.

Our study has several limitations. First, a retrospective, single-center design was used with a focus on a single determination of ANA at the time of IPF diagnosis. Second, an extended panel of guideline-recommended autoantibodies, such as the myositis panel, was not uniformly performed, as in a considerable number of cases, the diagnosis was made before publication of the recommendation for specific serologic testing in cases of suspected IPF [1, 2]. Third, we were unable to adjust the survival analysis for anti-fibrotic treatment because it was initiated at different times and for different durations during the study.

Further prospective multicenter studies evaluating a broader array of autoantibodies and responses to anti-fibrotic therapy are needed to fully explore the prognostic significance of ANA in individuals with IPF.

## Conclusion

A high ANA titer was associated with an increased risk of mortality in patients with IPF. Patients with IPF and a high ANA titer may benefit from heightened medical attention, including earlier consideration of anti-fibrotic therapy and referral for lung transplantation.

## Data Availability

The data supporting the findings of this study are available from the corresponding author upon reasonable request.
